# Qigong Training Positively Impacts Both Posture and Mood in Breast Cancer Survivors With Persistent Post-surgical Pain: Support for an Embodied Cognition Paradigm

**DOI:** 10.3389/fpsyg.2022.800727

**Published:** 2022-02-21

**Authors:** Ana Paula Quixadá, Jose G. V. Miranda, Kamila Osypiuk, Paolo Bonato, Gloria Vergara-Diaz, Jennifer A. Ligibel, Wolf Mehling, Evan T. Thompson, Peter M. Wayne

**Affiliations:** ^1^Laboratory of Biosystems, Institute of Physics, Universidade Federal da Bahia, Salvador, Brazil; ^2^Osher Center for Integrative Medicine, Harvard Medical School and Brigham and Women’s Hospital, Boston, MA, United States; ^3^Department of Physical Medicine and Rehabilitation, Harvard Medical School, Spaulding Rehabilitation Hospital, Boston, MA, United States; ^4^Zakim Center for Integrative Therapies and Healthy Living, Harvard Medical School, Dana Farber Cancer Institute, Boston, MA, United States; ^5^Department of Family and Community Medicine, Osher Center for Integrative Medicine, University of California, San Francisco, San Francisco, CA, United States; ^6^Department of Philosophy, University of British Columbia, Vancouver, BC, Canada

**Keywords:** posture, mood, breast cancer, embodied cognition, Qigong

## Abstract

Theories of embodied cognition hypothesize interdependencies between psychological well-being and physical posture. The purpose of this study was to assess the feasibility of objectively measuring posture, and to explore the relationship between posture and affect and other patient centered outcomes in breast cancer survivors (BCS) with persistent postsurgical pain (PPSP) over a 12-week course of therapeutic Qigong mind-body training. Twenty-one BCS with PPSP attended group Qigong training. Clinical outcomes were pain, fatigue, self-esteem, anxiety, depression, stress and exercise self-efficacy. Posture outcomes were vertical spine and vertical head angles in the sagittal plane, measured with a 3D motion capture system in three conditions: eyes open (EO), eyes open relaxed (EOR) and eyes closed (EC). Assessments were made before and after the Qigong training. The association between categorical variables (angle and mood) was measured by Cramer’s V. In the EO condition, most participants who improved in fatigue and anxiety scales also had better vertical head values. For the EOR condition, a moderate correlation was observed between changes in vertical head angle and changes in fatigue scale. In the EC condition, most of the participants who improved in measures of fatigue also improved vertical head angle. Additionally, pain severity decreased while vertical spine angle improved. These preliminary findings support that emotion and other patient centered outcomes should be considered within an embodied framework, and that Qigong may be a promising intervention for addressing biopsychosocially complex interventions such as PPSP in BCSs.

## Introduction

Both historical psychological theories and a growing body of experimental literature support the idea that emotional state can affect physical posture and that, conversely, physical posture can affect how people feel emotionally (Darwin, 1872; James, 1884; Riskind and Gotay, 1982; Ekman and Davidson, 1993; Oosterwijk et al., 2009; Michalak et al., 2014). For example, those who are clinically depressed have been observed to have a more stooped posture (Canales et al., 2010; Wilkes et al., 2017), and depression is commonly associated with somatic symptoms, including slumped posture (Buyukdura et al., 2011), muscle tension (Gupta, 2009), and pain (Nyboe Jacobsen et al., 2006; IsHak et al., 2018). These observations align with the field of embodied cognitive science, which proposes that the physical body plays a key role in cognitive processes (Di Paolo et al., 2017) and that mood should be considered to be embodied (Colombetti, 2017). Indeed, the acknowledgment that cognitive processes in the brain and the body’s corporeality and physiology are discernible but inseparable elements of an integrated living system–functioning within the context of an individual’s environment–has become a leading view in contemporary science and the philosophy of the body (Varela et al., 2017; Di Paolo et al., 2018; Ataria et al., 2021).

There is a growing body of literature from experimental studies that demonstrate bidirectional relationships between posture and psychological variables supporting their essential interdependence. Numerous short-term studies of experimentally induced mood states have also shown that behavior can influence posture (Oosterwijk et al., 2009). Conversely, short-term studies of experimentally manipulated posture have shown that body shapes can affect an individual’s emotional state (Weineck et al., 2020). Adopting a stooped, neutral, or upright posture has been shown to influence persistence (Riskind, 1983), stress (Kwon and Kim, 2015), interoceptive accuracy and feelings of power (Carney et al., 2010; Weineck et al., 2019, 2021) but see Elkjær et al. (2020). Furthermore, some studies have shown that manipulating body positions can have an effect on perception and emotional processing, such as the valence of memory (Riskind, 1983; Michalak et al., 2014). Stooped posture has recently been shown to impair recovery from negative mood and lead to more negative thoughts (Veenstra et al., 2017). However, much less is known about the longer-term impact of postural changes on mood (Osypiuk et al., 2018).

The interdependence of posture and affect may be especially apparent in chronic medical conditions that include complex interactions between trauma, pain, and impaired psychosocial function (Osborn and Smith, 2006; Martínez et al., 2018). Persistent post-surgical pain (PPSP) experienced by breast cancer survivors (BCS) exemplifies this constellation of morbidities. BCS PPSP is defined as a dull, burning, or aching pain most commonly felt in the chest, axilla, or upper extremity lasting at least 3 months post-surgery (Beyaz et al., 2016; Tait et al., 2018). BCS PPSP has a surprisingly high prevalence ranging from 25 to 60% (Miller et al., 2019). While underlying physiological mechanisms such as neuropathic or musculoskeletal are commonly implicated, the condition is appreciated to be biopsychosocially complex and likely less related to nociception (Moseley and Vlaeyen, 2015). BCS with diagnosed PPSP experience increased rates of anxiety and depression, fatigue, fear of recurrence, and poor body image (Kenyon et al., 2014). BCS PPSP has been associated with disability, and several studies have documented the negative effects of PPSP on activities of daily living, sleep, and overall quality of life (Gulluoglu et al., 2006; Kaya et al., 2010; Kenne Sarenmalm et al., 2014). Pain catastrophizing has been shown to increase the risk of developing PPSP in BCS (Belfer et al., 2013; Schreiber et al., 2013). Finally, posture itself has been shown to be adversely impacted by mastectomy (Malicka et al., 2010; Glowacka et al., 2016; Atanes Mendes Peres et al., 2017).

Because of the complex constellation of physical and psychosocial symptom elements experienced by cancer survivors, including BCS with PPSP, it has been hypothesized that optimal treatment strategies should be integrative and multi-modal, addressing concerns of the body, mind, and spirit (Carlson et al., 2017; Wayne et al., 2018; Osypiuk et al., 2020a). Qigong exercise represents one such approach. Qigong is a mind-body exercise originating in China which incorporates slow, coordinated movements, breath training, heightened somatic awareness, and mental focus and imagery (Klein, 2017). Recent systematic reviews have suggested that Qigong may be beneficial for improving overall quality of life, mood, sleep, and symptoms such as fatigue in cancer patients (Klein et al., 2016; Wayne et al., 2018; Kuo et al., 2021; Wang R. et al., 2021). In a pilot study conducted by our group—the parent study for the data presented here—we evaluated the impact of a 12-week Qigong intervention in BCS with PPSP. Findings reported elsewhere support that practicing Qigong can bring positive and clinically meaningful outcomes in multiple domains of physical and psychological function and quality of life (Osypiuk et al., 2020a,b). Building on these findings, in this study we specifically evaluated how practicing Qigong influences the inter-relationship between multiple aspects of posture and affect. Based on an embodied cognition framework, we utilized outcomes from our pilot study to evaluate longer-term bidirectional feedback loops between posture and affect that might impact the persistence of symptoms and recovery (Veenstra et al., 2017). Specifically, the aims of the present study investigate if Qigong-induced changes in body posture and affect are interdependent, such that changes in one might modulate the other. In this preliminary pilot study, our specific goals were aimed to assess the feasibility of objectively measuring posture, to preliminarily evaluate whether a relationship between posture and mood exists, and to explore design features for future research evaluating the impact of Qigong training on the relationship between posture and mood.

## Materials and Methods

### Study Design

This study is a single-arm, mixed-methods pilot clinical trial evaluating the effects of a 12-week QMBE intervention in breast cancer survivors (BCS) with PPSP. The main study involved collection of self-reported outcomes (e.g., pain, breast cancer symptoms, quality of life, exercise level) and objectively assessed functional outcomes (shoulder range of motion and grip strength), in combination with qualitative interviews assessed at both baseline and follow-up (Osypiuk et al., 2020a,b). Outcome measures were collected in person at baseline and post-intervention at the Motion Analysis Laboratory at Spaulding Rehabilitation Hospital. Posture was measured during quiet standing using a 3D motion capture system (Vicon, Oxford, United Kingdom). The study was approved by the Dana-Farber Cancer Institute (DFCI) Institutional Review Board.

### Participants

A total of 21 women with a history of stage 0—III breast cancer who had undergone surgical treatment and reported experiencing PPSP at least 3 months after completing surgery, chemotherapy, and/or radiation were enrolled in the study. Individuals were considered ineligible if they had any unstable chronic medical condition, were currently enrolled in physical therapy, exercised more than 240 min per week, or recently participated in QMBE, yoga, or Tai Chi classes on a regular basis.

### Intervention

The intervention practiced by study participants was based on the Eight Strands of the Brocades Qigong, which focuses on a sequence of eight movements that all engage the upper and lower extremities as well as the trunk (Kam-Chuen, 1991). The practice also incorporates elements such as focused attention, imagery, breath training, and relaxation. Courses were taught by experienced instructors. Participants were asked to attend one 1.25-h class per week for 12 weeks and to practice at home using a provided instructional video for 2–3 h per week.

### Outcomes

#### Clinical Measures

A subset of the collected self-reported clinical outcomes spanning a broad range of symptoms was selected to examine correlations with posture measurements. Pain was measured using the Brief Pain Inventory Short Form (BPI), a well validated 9-item measure of the severity and impact of pain on daily function (Keller et al., 2004; Mendoza et al., 2006). The Functional Assessment of Chronic Illness Therapy Fatigue Subscale (FACIT-F), a 13-item measure of the intensity of fatigue during the past 7 days, was used to assess fatigue (Cella et al., 2002). Self-esteem was assessed using the Rosenberg Self-Esteem Scale (RSE) (Rosenberg, 1965), anxiety and depression were measured using the Hospital Anxiety and Depression Scale (HADS) (Bjelland et al., 2002), and stress levels were measured using the Perceived Stress Scale (PSS) (Cohen et al., 1983). Exercise self-efficacy was measured with the 13-item Self-Efficacy for Exercise scale (SEE) (Resnick and Jenkins, 2000), which measures confidence in one’s ability to exercise in the face of barriers.

#### Posture Data Acquisition

A set of 10 reflective markers was positioned on the following anatomical landmarks: both ear lobes, right and left acromion, 7th cervical vertebra (C7), 8th thoracic vertebra (T8), right and left posterior superior iliac spine (PSIS), right and left calcaneus. Posture was measured during a 40-s trial of quiet standing for each of three conditions: (1) eyes open (EO), (2) eyes open and relaxed (EOR)—the participants were asked to look forward and stay as relaxed as possible—(3) eyes closed (EC). Participants were instructed to stand as still as possible, facing forward. During the EO condition, they were asked to “stand up straight,” while in the EOR condition they were informed that they could “relax and stand naturally”; participants were asked to remain in the same position as EOR but to close their eyes for the EC condition. The EOR and EC conditions were intended to decrease the focus of the participants on their postures and thereby encourage the most natural posture possible. Videos were recorded using a 10-camera marker-based motion capture system (Vicon, Oxford, United Kingdom).

### Posture Parameters

The reflective markers’ time series were used to compute angles between body segments that characterize key features of posture using a Matlab (Mathworks, Massachussets, United States) custom script. Specifically, we computed the vertical spine (VertSpi) angle and the vertical head (VertHead) angles in the sagittal plane from a lateral view as shown in Table 1. Postural angles were computed using standard techniques in biomechanics (Uchida and Delp, 2020).

**TABLE 1:** Description of angle names and components.

Plane	Angle name	Anatomical landmarks	Figure	Interpretation
Sagittal	Vertical Head (VertHead)	Right ear lobe, C7 and vertical plane	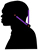	Lower is better
Sagittal	Vertical Spine (VertSpi)	C7, T8 and the middle point between both EIPS computed with MatLab	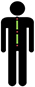	Higher is better

After computing the angles’ time series, the mean and standard deviation (SD) were derived for each angle. Higher values for the vertical spine (VertSpi) angle were interpreted as better posture, indicating that the spine was straighter. Conversely, lower values of the vertical head (VertHead) angle were interpreted as better posture, indicating a lower degree of head anteriorization.

Since the time series of the VertSpi and VertHead angles were available, it was possible to measure the variability in the angular displacement measures and set a threshold to determine if the longitudinal changes were significant. Accordingly, the variable outcomes were discretely and categorically defined into “better” and “worse” posture when there was improvement or worsening beyond the intra-individual standard deviation of the baseline fluctuation, and no change when the follow-up mean was within the baseline fluctuations. The range of the threshold values for each condition and angle for all participants were: EO—VerHead: 1.76°, VerSpi: 0.8°; EOR—VerHead: 0.66°, VerSpi: 0.29°; EC—VerHead: 1.93°, VerSpi: 0.41°. The results of mood measures (PSS, SEE, HADS, RSE, FACIT-F, BPI) were also categorized as “better” or “worse” by comparing subjects’ scores at follow-up with their scores at baseline. If the subject had an improvement in the fatigue scale, for example, it was categorized as “better mood.”

### Statistical Analysis

Descriptive statistics were used to summarize subjects’ baseline characteristics. Outcome measures were scored according to standardized algorithms. The association between the categorical variables (angle and mood) was measured by the Cramer’s V. This measurement reflects the degree of association between two categorical variables and is computed based on a modified Chi-square statistic, the sample size, and degrees of freedom (Field, 2000). Since the Cramer’s *V*-values range between 0 and 1, the Pearson correlation value interpretation was used for this purpose. Thus, values between 0 and 0.499, 0.500 and 0.699, 0.700 and 0.899, and 0.900 and 1.000 were considered as weak, moderate, strong, and very strong correlations, respectively (Mukaka, 2012). These values filled the heat maps which illustrate the association between mood and posture variables. The colors ranged in a gradient from white (weakest correlation) to red (strongest correlation). The level of statistical significance considered in this study is 5% and no multiple comparisons adjustment was made.

## Results

**FIGURE 1:**
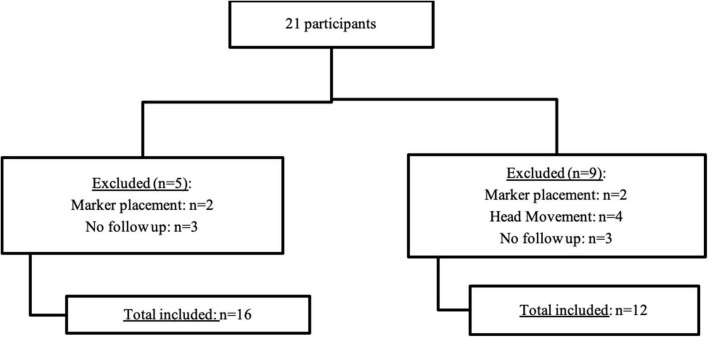
Flow chart of number of participants in each angle analysis.

We enrolled 21 participants in total, however, three participants were lost to follow-up. There were also two marker placement errors (follow up evaluation markers were positioned incorrectly) that compromised data reliability for all conditions. We excluded these participants from estimates of postural angles requiring these specific markers. An additional four participants in the EOR condition were excluded because they moved the head during the assessment, compromising the analysis (Figure 1). For subsequent analyses we had a total of 16 patients for EO and EC conditions and 12 subjects for the EOR condition.

### Eyes Open

**FIGURE 2:**
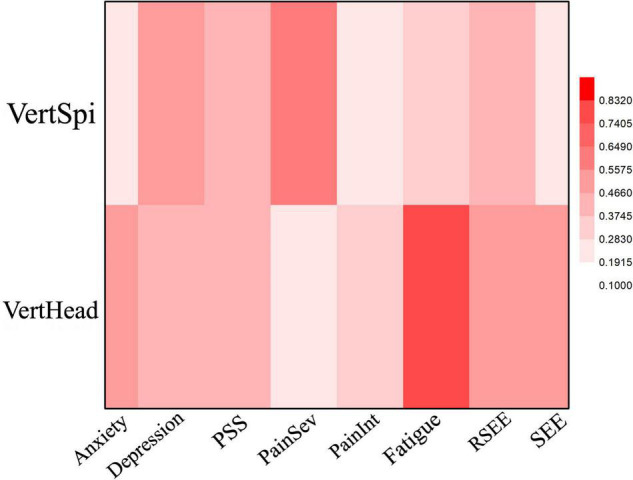
Heat Map of the correlation between mood and posture angles for the EO condition. Dep, Depression; PainSev, Pain Severity; PainInt, Pain Interference.

Most participants who improved in fatigue and anxiety scales also improved VertHead values. Thirteen subjects improved fatigue and from these, nine improved the VertHead angle, three got worse and one did not change. Three participants had worse fatigue at follow-up and none of them changed angle values before and after intervention. Similarly, nine participants improved anxiety scores, and of these, seven improved their VertHead angle and two got worse. Of the six subjects who reported increased anxiety, four did not change posture or exhibited poorer posture. Figure 2 and Table 2 show a strong (Cramer’s *V* = 0.83, *p* = 0.004) and moderate (Cramer’s *V* = 0.55, *p* = 0.04) association between vertical head angle and fatigue and anxiety, respectively.

**TABLE 2:** Cramer’s *V* and *p*-values for the correlations between mood and posture angles for the Eyes Open (EO) condition.

	*p*-value								
	
	Anxiety	Dep	PSS	PainSev	PainInt	Fatigue	RSEE	SEE
VertHead	0.04*	0.3	0.16	0.6	0.6	0.004*	0.08	0.1
VertSpi	0.66	0.1	0.22	0.08	0.84	0.33	0.24	0.6

**Cramer’s V**								

VertHead	0.55*	0.4	0.46	0.25	0.29	0.83*	0.5	0.5
VertSpi	0.27	0.5	0.42	0.56	0.21	0.37	0.41	0.3

Dep, Depression; PainSev, Pain Severity; PainInt, Pain Interference. *Moderate or high Cramer’s V correlation that is also statistically significant.

### Eyes Open Relaxed

**FIGURE 3:**
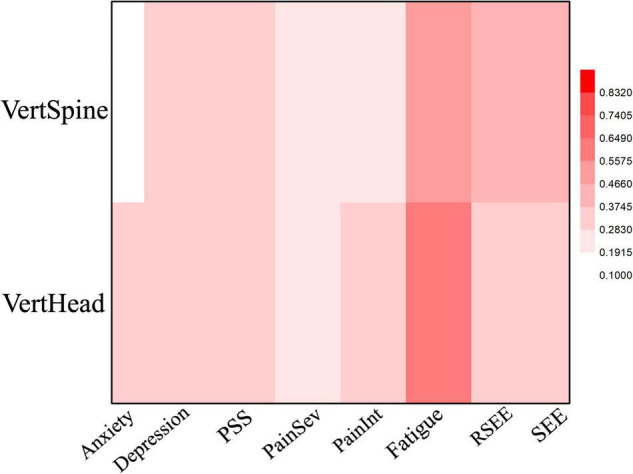
Heat Map of the correlation between mood and posture angles for the EOR condition. Dep, Depression; PainSev, Pain Severity; PainInt, Pain Interference.

We also observed an association between VertHead angle and fatigue scores in the EOR testing condition (Cramer’s *V* = 0.62, *p* = 0.04) (Figure 3 and Table 3). Out of the thirteen subjects that reported improved fatigue scores, ten improved, two worsened, and one did not change VertHead posture. For the three participants that reported worse fatigue, one did not change the VertHead posture and two exhibited poorer posture.

**TABLE 3:** Cramer’s V and *p*-values for the correlations between mood and posture angles for the Eyes Open Relaxed (EOR) condition.

*p*-Value								
	
	Anxiety	Dep	PSS	PainSev	PainInt	Fatigue	RSEE	*SEE*
VertHead	0.4	0.6	0.59	0.63	0.57	0.04*	0.43	0.41
VertSpi	0.9	0.44	0.6	0.63	0.64	0.17	0.33	0.2

**Cramer’s V**								

VertHead	0.36	0.29	0.3	0.24	0.3	0.62*	0.35	0.33
VertSpi	0.18	0.34	0.29	0.24	0.28	0.47	0.38	0.45

Dep, Depression; PainSev, Pain Severity; PainInt, Pain Interference. *Moderate correlation that is statistically significant.

### Eyes Closed

**FIGURE 4:**
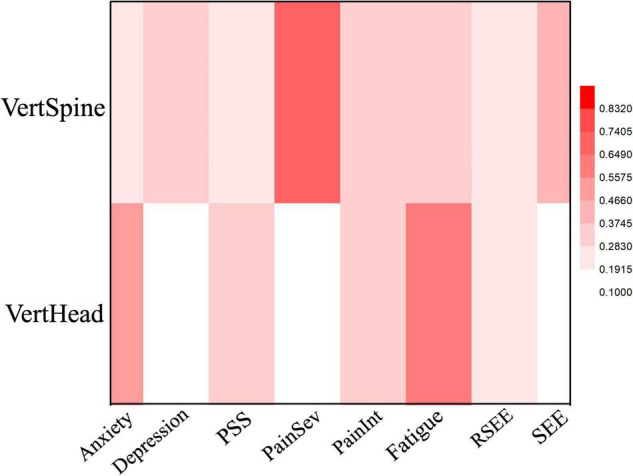
Heat Map of the correlation between mood and posture angles for the EC condition. Dep, Depression; PainSev, Pain Severity; PainInt, Pain Interference.

As in both EO and EOR conditions, most of the participants in the EC testing condition that reported improved fatigue scores also improved their VertHead angle (Figure 4 and Table 4). Of the 13 subjects that improved fatigue, 10 improved the VertHead posture and 3 had worse posture. All three participants that had reported worse fatigue also exhibited worse VertHead posture. There was a significant but moderate association between the variables (Cramer’s *V* = 0.62, *p* = 0.01). Also noteworthy under the EC condition was a strong correlation (Cramer’s *V* = 0.74, *p* = 0.01) between pain severity and VertSpine angle. Of the 14 subjects who reported improvement in pain severity (i.e., less pain), 10 exhibited improved VertSpine posture while four exhibited worse posture. Of the two subjects that reported more pain in the follow up evaluation, one did not exhibit a change in VertSpine posture and the other exhibited worse posture.

**TABLE 4:** Cramer’s V and *p*-values for the correlations between mood and posture angles for the Eyes Closed (EC) condition.

*p*-value								
	
	Anxiety	Dep	PSS	PainSev	PainInt	Fatigue	RSEE	SEE
VertHead	0.16	0.92	0.42	0.7	0.34	0.01*	0.73	0.51
VertSpi	0.84	0.36	0.64	0.01*	0.6	0.33	0.75	0.27

**Cramer’s V**								

VertHead	0.48	0.1	0.33	0.1	0.36	0.62*	0.2	0.16
VertSpi	0.21	0.37	0.28	0.74*	0.29	0.37	0.24	0.4

Dep, Depression; PainSev, Pain Severity; PainInt, Pain Interference. *Moderate and high correlation that are statistically significant.

## Discussion

The main goal of this pilot study was to assess the feasibility of characterizing the relationship between posture and mood in BCS with PPSP engaged in a 12-week Qigong mind-body training program. We determined that it is feasible to reliably measure key features of upper body posture using a marker-based kinematic system. Moreover, our preliminary findings support that over the course of 12 weeks of Qigong training, improvements in VertHead and VertSpine postures were associated with improvements in cancer-related anxiety and fatigue, and under eyes closed conditions, reduced pain severity. Along with already published quantitative and narrative results from this pilot study that also highlight a more embodied perspective of breast cancer survivors following Qigong training (Osypiuk et al., 2020a,b), these findings support the importance of more in-depth research elucidating the effects of multimodal mind-body practices on the interdependence of posture and psychological well-being in clinical conditions.

The interdependence of human posture and affect is widely accepted as an integral component of an embodied cognition framework (Niedenthal, 2007), and a growing body of experimental studies supports that short-term (i.e., minutes to hours) experimental manipulations of posture or facial expression are associated with changes in mood, and conversely short-term induced changes in mood can alter body shapes and movement patterns (Osypiuk et al., 2018; Carney, 2020; Elkjær et al., 2020). However, very few clinical studies have evaluated the longer-term impact of interventions on these interdependences for mental and physical health and well-being in clinical populations. Michalak and colleagues conducted one of the few longitudinal clinical interventions exploring associations between affect, posture and gait characteristics (Michalak et al., 2011). At baseline, 23 formally depressed outpatients (in remission for Major Depressive Disorder) were compared to individuals with no prior diagnosis of depression. Despite being in remission, compared to healthy controls, formerly depressed individuals exhibited significantly reduced walking speed and vertical movements of the upper body—-both shown in prior studies to be associated with depression (Michalak et al., 2009; Gross et al., 2012; Rosario et al., 2014; Wang Y. et al., 2021). In Michalak’s study (Michalak et al., 2011) the vertical head movement measured the vertical displacement of the head marker. A small but non-significant trend toward a reduced vertical displacement of the head posture was also observed in individuals with prior depression. Using a pre-post design, all formerly depressed patients were then exposed to a course of mindfulness-based cognitive behavioral therapy (MBCT); 8 weekly group sessions; 2.5 h/session)—which like Qigong, includes training in body awareness (Fissler et al., 2016). After MBCT, walking speed and lateral swaying movements of the upper body were normalized and a trend toward normalization of vertical head movements was also observed. Unlike our study, no average pre-post changes in neck angle were observed, but there was a small and statistically non-significant correlation between changes in depression and both posture and mindfulness measured with the Mindfulness Attention and Awareness Scale (MAAS) (Pearson correlations *r 0*.26 and 0.27, respectively). The authors concluded that MBCT has a normalizing effect on gait patterns, thus displaying not only cognitive, but also “embodied” effects. Of note, our study also observed a positive association between improved VertHead angles and self-reported depression scores in both eyes-open conditions, however, these trends were not statistically significant.

Under all conditions, we observed a positive association between improved VertHead angles and self-reported anxiety, with a statistically significant and moderate effect size for the eyes open condition. This finding aligns with a growing body of short-term experimental studies linking posture and anxiety (Lipnicki and Byrne, 2008; Nair et al., 2015; Asadi-Melerdi et al., 2020). For example, one study among adults with depression showed that a single session of adopting an upright posture led to acute reductions in anxiety and negative affect, compared with sitting in a neutral position (Wilkes et al., 2017). A more recent two-arm longitudinal study of healthy college students reported that adopting either experimentally prescribed powerful or neutral postures for 2 weeks led to reduced trait anxiety (Weineck et al., 2020). Interestingly, improvements in trait anxiety in both interventions paralleled improvements in interoceptive awareness. This two-arm study did not include either an exaggerated contracted or a normal posture control intervention, therefore limiting the study’s ability to link specific postural characteristics to affective states. However, findings from a recent systematic review suggest that both expansive and neutral postural positions are associated with benefits to affect, when compared to contracted postures (Elkjær et al., 2020). Collectively, these findings suggest that embodied interventions, including Qigong, that encourage adopting an open or expansive bodily posture (e.g., greater VertHead) whilst maintaining a self-focus, may help to reduce anxiety and more generally improve affective state (Osypiuk et al., 2018). Our observations of the interdependence of upper body posture and affect is also supported by physiological studies showing links between stress-induced changes in neck and upper trunk tone assessed with electromyography and dysregulated autonomic tone assessed with indices of heart rate variability (Kristiansen et al., 2009; Kang et al., 2012; Santos-de-Araújo et al., 2019; Scheer et al., 2021). The interdependence of posture and anxiety is exhibited in a rich body of observational and experimental studies linking fall-related anxiety and acrophobia (fear of heights), neuromusculoskeletal physiology, and whole-body postural control (Adkin and Carpenter, 2018). This latter body of research supports a broader leveraging of the embodied cognitive framework to include studies linking affective states to whole body dynamic postural control, both in laboratory settings using camera-based motion capture systems such as the Vicon system used in this study (Michalak et al., 2009, 2015; Kumar et al., 2021), as well in real world environments using wearable sensors that may inform dimensions of embodied cognition while individuals engage in practical everyday activities (Shull et al., 2014; Benson et al., 2018; Nouredanesh et al., 2021).

An intriguing finding from this study was the strong association between improvements in Vert-Head posture and cancer-related fatigue. Fatigue is one of the most common, persistent, and distressing side effects of cancer and cancer treatment, disrupting multiple aspects of quality of life while also contributing to reduced survival (Bower, 2014; Berger et al., 2015). Cancer-related fatigue is typically more severe and less likely to be relieved by rest than general fatigue. Emerging research suggests that levels of fatigue in cancer patients are associated with an array of psychosocial and genetic factors, with underlying biology involving changes in multiple markers of inflammation (Bower, 2014; Berger et al., 2015). Multimodal mind-body interventions, including Qigong and Tai Chi, have shown promise for alleviating cancer-related fatigue (Wayne et al., 2018; Liu et al., 2021), possibly through their impact on inflammation (Irwin et al., 2014, 2015; Bower and Irwin, 2016; Kinney et al., 2019). The evidence for links between posture and fatigue in cancer has not been previously described. However, some experimental evidence supports an interrelationship between fatigue, inflammation and postural control. Utilizing a “sickness” model in which lipopolysaccharide (LPS) injection is used to induce a reliable and robust acute inflammatory response, multiple studies have reported an association between systemic cytokine levels and fatigue (Lasselin et al., 2020a). Moreover, LPS exposure and higher cytokine levels are also associated with slower walking, wider strides, less arm extension, less knee flexion, and a more downward-tilting head while walking (Lasselin et al., 2020b). One offered evolutionary sociobiological interpretation of these findings is that the illness related movement patterns contain information about an individual’s health status, which could provide an advantage from a disease-avoidance perspective, and/or could be useful in identifying individuals in need of care (Lasselin et al., 2020b). These finding further support the value of studying links between whole body postural control and psychoneuroimmunological processes underlying embodied behavior.

A key goal of Qigong and other practices that integrate mind-body training is to enhance self-awareness, at least in part through heightened interoception and non-judgmental appraisal of, and increased reliance on and trust in, experienced physical sensations (Mehling et al., 2011; Farb et al., 2015; Weng et al., 2021). The findings from this study appear to indicate that improved interoception and mindful self-awareness resulting from Qigong training contributed to shifts in perceived pain, posture and affect, along with increased conscious awareness of the interdependencies between these sensory experiences. This is supported by significant improvements among participants in this study following Qigong training in the Multidimensional Assessment of Interoceptive Awareness (MAIA) (previously reported) (Mehling et al., 2018), including in sub-domains related to Noticing (awareness of uncomfortable, comfortable, and neutral body sensations), Emotional Awareness (awareness of the connection between body sensations and emotional states), Self-Regulation (ability to regulate distress by attention to body sensations) and Trusting (experience of one’s body as safe and trustworthy) (Osypiuk et al., 2020b). Increased conscious awareness of the interdependencies between postural and affective dimensions was also supported by narrative reports shared by study participants (previously reported; Osypiuk et al., 2020a). Two primary emergent qualitative themes identified included: (1) Qigong enabled participants to reconnect mind and body and lessened their pain; and (2) QMBE enabled BCS to make peace with their bodies, fostering acceptance and renewed confidence in their bodies. These are reflected in the following representative quotes:

“How you feel about your body is a challenge after you’ve had breast cancer. … [But] mind and body have to be interconnected. All of it together [in Qigong] relaxes you and helps you stretch out a little bit, calm you down, help you think about your body in a different way, and trust your body to get inside yourself in a different way. It doesn’t mean you’re not going to get cancer again, but it could mean that you’re more at peace with your own body.”

“I feel that if I can get the body and the mind on the same track together, then it’s not defining myself by, “Oh! I’m breast cancer four generations and I’m stage 1A and I’m TNBC,” and all these labels, these quantitative markers that says nothing about…all the other ways I’d like to define myself. … This is about the big picture, healing between mind and body, improving the constellation of different symptoms.”

Qigong’s multimodal approach integrating movement, breath training, and mindfulness of both somatic sensations and thoughts/mental states appears to increase the awareness of the complex interactions of body states with cognitive and emotional processing. Such heightened awareness might not only assist patients in recognizing habitual and inefficient musculoskeletal patterns (e.g., excessive neck tone and forward-tilting head posture) or negative self-referential thoughts, but also in recognizing and disentangling potential habitual maladaptive feedback loops between dysfunctional movement patterns and negative emotional states and thoughts. A similar embodied cognitive framework was used by Michalak and colleagues for interpreting the impact of MBCT in remission for MDD. Namely, mindful awareness of deviant gait and posture may minimize the chance of an embodied depressed phenotype lingering in the body and de-escalate mind/body viscous cycles that lead to depressive relapse (Michalak et al., 2011).

The findings from our study support that enhanced self-awareness resulting from Qigong training may have beneficial clinical effects in BCS (Huang et al., 2021; Meng et al., 2021). Along with other findings from earlier studies of mind-body interventions, these findings suggest a broader clinical relevance of theoretical frameworks emphasizing the embodied nature of emotion (Damasio, 1995; Niedenthal et al., 2005; Colombetti, 2014) and the enactivist conception of the body (Di Paolo and Thompson, 2014; Varela et al., 2017). According to these embodiment theories, the bi-directional interaction between cognitive and affective results in emotional states and traits affecting somato-visceral and motor systems, and conversely, bodily states affect the processing of emotional information (Colombetti, 2014). Cognition and emotion form a unitary interactive system, in which physiology and psychology are distinguishable but inseparable (Pessoa, 2013). Numerous other bodies of research support the embodiment model (Tantia, 2020). For example, research focused on gestures has highlighted the coordination of talk with bodily action, demonstrating the multimodal nature of communication with its expression of emotions (Goldin-Meadow, 2003; McNeill, 2005). Research on visual perception has shown that self-generated bodily movement directly affects how and what we perceive (Noe, 2005; Wexler and van Boxtel, 2005). In general terms, one of the principle aims of embodied cognitive science is to devise explanatory models that specify how the body, brain, and environment mutually interact and make up a larger dynamic system in which the organism constantly adapts to the demands of a changing environment (Thompson, 2007; Chemero, 2011).

Intriguingly, these modern theories align with traditional dialectical theories of contemplative mind-body practices that emphasize the co-creation of body postures and mental states (Osypiuk et al., 2018). Borrowing from Zen Buddhist teacher Shunryu Suzuki’s first words on posture instruction for meditation in his classic book, *Zen Mind, Beginner’s Mind*, “These forms are not the means of obtaining a right state of mind. To take this posture is itself to have the right state of mind” (Suzuki, 1970). Similarly, both contemporary and traditional mind-body practitioners insist that finding bi-directional interactions between mind and physical body in research studies points towards a deeper understanding of the role of the body in the lived experience and a unitary conceptualization of body and mind (Mehling et al., 2011). These mind-body practitioners describe their interventions (including Qigong) as enhancing interoceptive and proprioceptive bodily awareness, which may be conceptualized as a socially influenced developmental process connecting body schema and body image and creating the sense of the minimal and implicit self (Drysdale and Tsakiris, 2021).

A practical goal of this study was to evaluate the feasibility of obtaining precise and reliable measures of various components of standing posture in a laboratory setting. Using a camera-based motion capture system, we confirmed it is feasible to characterize two key parameters of posture, Vert-Head and Vert-Spine. Taking advantage of the temporal nature of kinematic estimates, we only included pre-post intervention changes that exceeded the variability observed within a testing session. Future studies should evaluate the feasibility and relevance of other elements of postures; for example, the degree of concavity of the chest is likely associated with shame and pride. We also employed two eye-open testing conditions (i.e., participants were instructed to “stand up straight,” vs. “relax and stand naturally”) to evaluate potential impact of conscious test performing bias. While trends in both conditions were qualitatively similar, a growing body of research supports bias associated with externally motivated behaviors and white-coat effect during postural control testing (Van Ancum et al., 2019; Orcioli-Silva et al., 2021). Future larger studies are needed to inform the value of comparing both eyes-open conditions. For this first proof-of-concept study, we used a camera-based motion capture system which is costly and requires relying on personnel with significant technical training. We chose this over simpler goniometric approaches to minimize bias associated with examiners’ judgment, inter-examiner variability, and study postural self-consciousness during assessment. However, if in future studies key postures are identified that can be measured with good precision and minimal bias using goniometric approaches, this could serve as a less expensive and practical measure for clinical studies. Other emerging technologies based on video analysis using advance machine learning techniques should also be considered (Cao et al., 2021). Finally, to better inform dimensions of embodied cognition in real world environments, future studies should also consider using wearable sensors (Poitras et al., 2019) that may inform dimensions of embodied cognition while individuals engage in socially enriched and practical everyday activities over extended periods of time (Shull et al., 2014; Benson et al., 2018; Nouredanesh et al., 2021).

## Limitations and Future Directions

Our study has several limitations. Interpretation of the clinical effects should be considered preliminary, as this was not a randomized trial and our sample size was small. The generalizability of our study is also limited as the participants were a self-selected group who had an interest in mind-body therapies. Subject selection was not based on pain levels; the participants had relatively low levels of pain at baseline, possibly leading to a floor effect. However, while pain ratings were low, qualitative interviews revealed that even low levels of pain cause significant distress; a more multidimensional approach to measuring the extent of this burden may be more appropriate than a simple pain scale. The pain participants experienced was heterogeneous in terms of type and location. In future studies, PPSP should be more specifically defined and women with clinically significant levels of pain (> 3/10) should be selected. Our measures were obtained under non-reactive conditions, thus we were less likely to see large effect sizes (Smith et al., 1980). Future studies might consider conditions that provoke emotional states, include posing in emotionally provocative postures, or both.

## Data Availability Statement

The original contributions presented in the study are included in the article/supplementary material, further inquiries can be directed to the corresponding author.

## Ethics Statement

The studies involving human participants were reviewed and approved by Dana-Farber Cancer Institute Institutional Review Board. The patients/participants provided their written informed consent to participate in this study.

## Author Contributions

PW conceived of and designed the study. AQ and PW created the first draft of the manuscript. KO, ET, WM, JL, PB, JM, and GV-D contributed critically important intellectual content. PB, JM, GV-D, and AQ oversaw measurement and analysis of posture data. All authors contributed to manuscript revision, read, and approved the submitted version.

## Conflict of Interest

PW was the founder and sole owner of the Tree of Life Tai Chi Center. PW’s interests were reviewed and managed by the Brigham and Women’s Hospital and Partner’s HealthCare in accordance with their conflict of interest policies. The remaining authors declare that the research was conducted in the absence of any commercial or financial relationships that could be construed as a potential conflict of interest.

## Publisher’s Note

All claims expressed in this article are solely those of the authors and do not necessarily represent those of their affiliated organizations, or those of the publisher, the editors and the reviewers. Any product that may be evaluated in this article, or claim that may be made by its manufacturer, is not guaranteed or endorsed by the publisher.
